# A case of pancreatic mucinous cystadenocarcinoma with malignant ascites without recurrence for more than 8 years after surgery

**DOI:** 10.1007/s12328-022-01639-z

**Published:** 2022-05-11

**Authors:** Shun Tezuka, Makoto Ueno, Satoshi Kobayashi, Taito Fukushima, Ryuji Nasu, Kota Washimi, Naoto Yamamoto, Soichiro Morinaga, Manabu Morimoto, Shin Maeda

**Affiliations:** 1 Department of Hepatobiliary and Pancreatic Medical Oncology, Kanagawa Cancer Center, 2-3-2 Nakao, Asahi-ku, Yokohama City, 241-8515 Japan; 2 Department of Clinical Laboratory, Kanagawa Cancer Center, Yokohama, Japan; 3 Department of Pathology, Kanagawa Cancer Center, Yokohama, Japan; 4 Department of Gastrointestinal Surgery, Kanagawa Cancer Center, Yokohama, Japan; 5 Department of Gastroenterology, Yokohama City University Graduate School of Medicine, Yokohama, Japan

**Keywords:** Mucinous cystadenocarcinoma, Mucinous cystic neoplasm, Cystic neoplasm of pancreas, Distal pancreatectomy

## Abstract

Mucinous cystadenocarcinoma (MCAC) with malignant ascites is rare. We report a case of a 28-year-old woman who presented with epigastric pain. The ascites in the Douglas fossa was identified at a nearby gynecology clinic. Computed tomography showed a multiloculated cystic lesion (9.5 × 6.4 cm) in the tail of the pancreas, which was diagnosed as mucinous cystic neoplasm on imaging. Staging laparoscopy was performed, and rapid cytology of ascites revealed adenocarcinoma, leading to a diagnosis of unresectable MCAC. Subsequently, combination chemotherapy with gemcitabine plus S-1 was initiated. Although there were no remarkable changes in the imaging findings, the peritoneal dissemination node was not consistently recognized in any of the imaging findings, and distal pancreatectomy was performed. A peritoneal dissemination node was not observed in the laparotomy findings, but the peritoneal lavage cytology was positive. The postoperative pathological result was non-invasive MCAC, and the ascites was suspected to be caused by cyst rupture. The patient has been recurrence-free, including the reappearance of ascites, for > 8 years after adjuvant therapy with S-1. Although careful follow-up will be required in the future, the very good prognosis in this case suggests that MCAC with malignant ascites without obvious peritoneal dissemination should be considered for surgical resection.

## Introduction

Pancreatic mucinous cystic neoplasm (MCN) is a rare cystic neoplasm that is typically located in the pancreatic body or tail of middle-aged women [1]. In 2000, the World Health Organization (WHO) defined MCN based on the presence of ovarian stroma on histopathology [2]. MCN is classified as mucinous cystadenoma, mucinous cystic neoplasms with moderate dysplasia, and non-invasive and invasive mucinous cystadenocarcinoma (MCAC), according to the degree of dysplasia [3, 4]. According to the WHO 2000 report after the WHO 2000 classification, of those who underwent surgical resection of a pancreatic cystic neoplasm, the frequency of MCN and MCAC was 21% and 7.9%, respectively [5].

When MCAC is localized in the pancreas, surgical resection is recommended and mostly associated with a favorable prognosis [6]. Palliative chemotherapy is recommended for unresectable MCAC [7]; however, there have been few case reports including chemotherapy for unresectable MCAC [8, 9], and no large-scale retrospective or prospective studies have been reported. In clinical practice, unresectable MCAC is treated with chemotherapy, similar to pancreatic ductal carcinoma.

We report the case of a patient with MCAC with malignant ascites who underwent surgical resection after combination chemotherapy with gemcitabine plus S-1 (GS). Although surgical resection is not indicated for pancreatic ductal carcinoma with malignant ascites, in this case, surgical resection was performed because of the absence of peritoneal dissemination and the young age. As a result, the patient has had no recurrence, including no reappearance of ascites for more than 8 years after postoperative adjuvant therapy.

## Case report

**Fig. 1:**
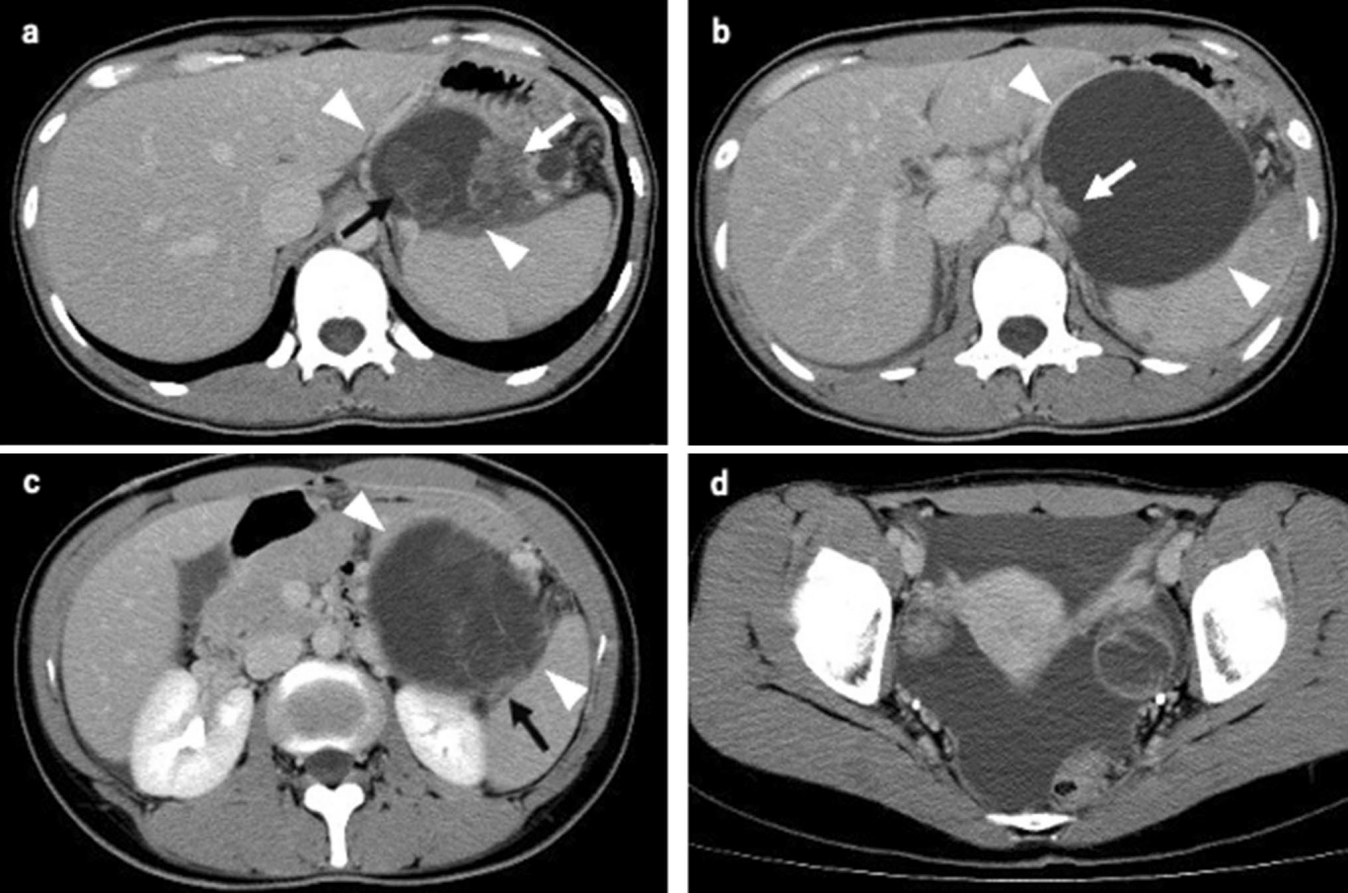
A 28-year-old woman with a mucinous cystic neoplasm of the pancreas. Contrast-enhanced computed tomography shows a well-defined 9.5 × 6.4 cm multilocular cystic lesion (white arrowhead) in the tail of the pancreas and ascites in pelvis. The cystic lesion had a cystic component (black arrow) and a mural nodule (white arrow) along the cyst wall

A 28-year-old woman had occasionally presented epigastric pain. Approximately 10 months after the onset of the epigastric pain, the patient visited a nearby gynecology clinic because of abnormal vaginal bleeding. Transvaginal ultrasonography at the clinic revealed ascites in the Douglas fossa, and she was referred to the gynecology department of a nearby university hospital. Douglas fossa puncture for the cytology of ascites was performed, and adenocarcinoma was suspected (Papanicolaou class IIIb). The gynecological examination revealed no abnormal findings in the uterus and ovary; however, the computed tomography (CT) scan revealed ascites in the pelvis and a well-defined 9.5 × 6.4 cm multilocular cystic lesion in the tail of the pancreas (Fig. 1). The cystic lesion had a cystic component ("cyst in cyst") and a mural nodule along the cyst wall, which was diagnosed as an MCN upon imaging. Staging laparoscopy was performed and a rapid cytology of the ascites revealed adenocarcinoma, leading to a diagnosis of unresectable MCAC. Fluid analysis showed elevated carcinoembryonic antigen (CEA) (328.1 ng/mL).

**Fig. 2:**
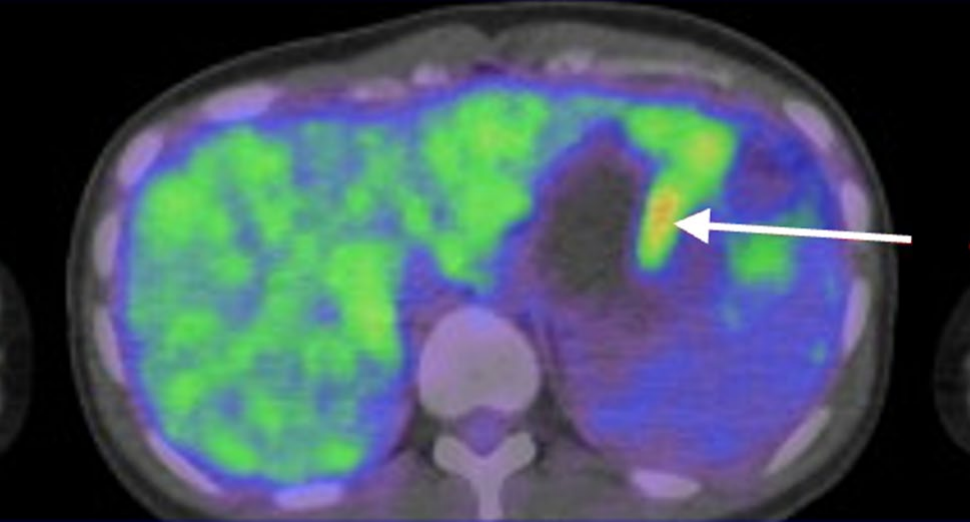
Positron emission tomography–computed tomography demonstrated 18F-fluorodeoxyglucose uptake only in the solid component (white arrow) of the pancreatic tail cyst, and maximum standard uptake values (SUVmax) was 2.94

**Fig. 3:**
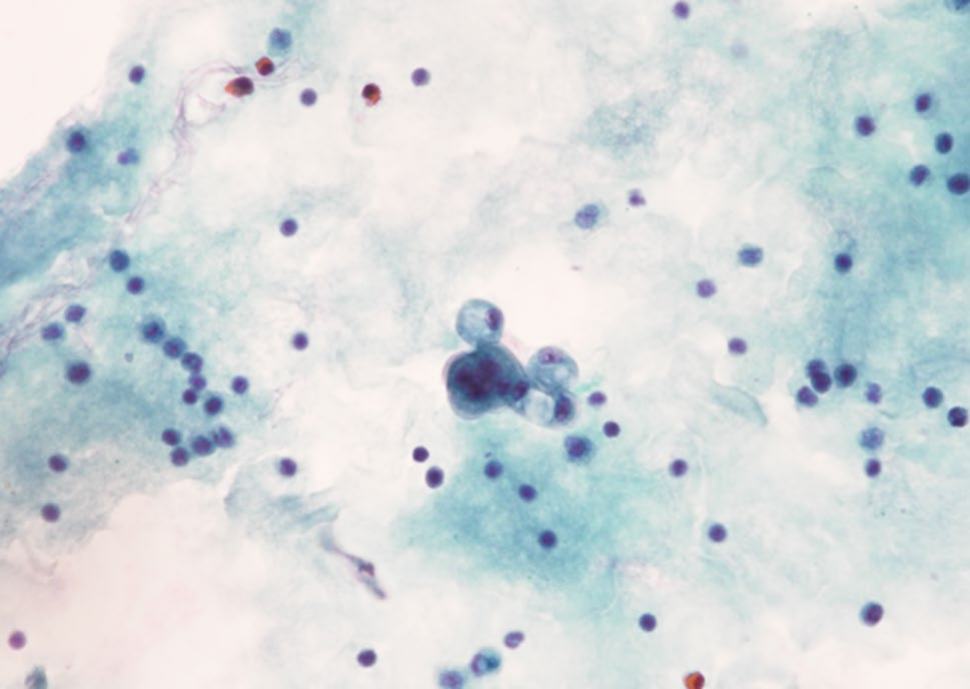
Ascites which existed on the liver surface was punctured, and the result of the cytology was adenocarcinoma

Subsequently, the patient was referred to our institution for further treatment. She had no previous medical history, including pregnancy. On physical examination, a soft, fist-sized mass was palpable in her left upper abdomen, with no abdominal pain at that time. Laboratory tests showed no abnormal findings, and bone marrow, liver, and kidney functions were normal. The values of serum tumor markers, CEA and carbohydrate antigen 19–9 (CA19-9), were 1.0 ng/mL and 30.7 U/mL, respectively. The cancer board with the department of surgery was carried out, and the diagnosis reached at our institution was also unresectable MCAC. Subsequently, combination chemotherapy with 1,000 mg/m^2^ of gemcitabine (GEM) on days 1 and 8 plus 100 mg/day of S-1 (an oral fluoropyrimidine) from days 1 to 14, repeated every 3 weeks per course, were initiated. Due to grade 4 neutropenia during the first course, the GEM dose was reduced to 80% from the second course. At the end of the sixth course (4.5 months later), the best overall response to chemotherapy on the Response Evaluation Criteria in Solid Tumors version 1.1 was stable disease on contrast-enhanced CT. Furthermore, although ascites was still observed, a slight decrease in serum CA19-9 was observed (from 30.7 to 9.9 U/mL). Positron emission tomography–CT (PET–CT) that was performed to re-evaluate the feasibility of surgery, demonstrated 18F-fluorodeoxyglucose uptake only in the solid component of the pancreatic tail cyst, and the maximum standard uptake value (SUVmax) was 2.94 (Fig. 2). Ascites on the liver surface was punctured, and the cytology result remained adenocarcinoma (Fig. 3). However, ascites was suspected to be caused by the rupture of the cyst, because the peritoneal dissemination node was not recognized in any of the imaging findings. Finally, considering the interpretation of the ascites and her young age, surgical resection was carried out by the cancer board.

**Fig. 4:**
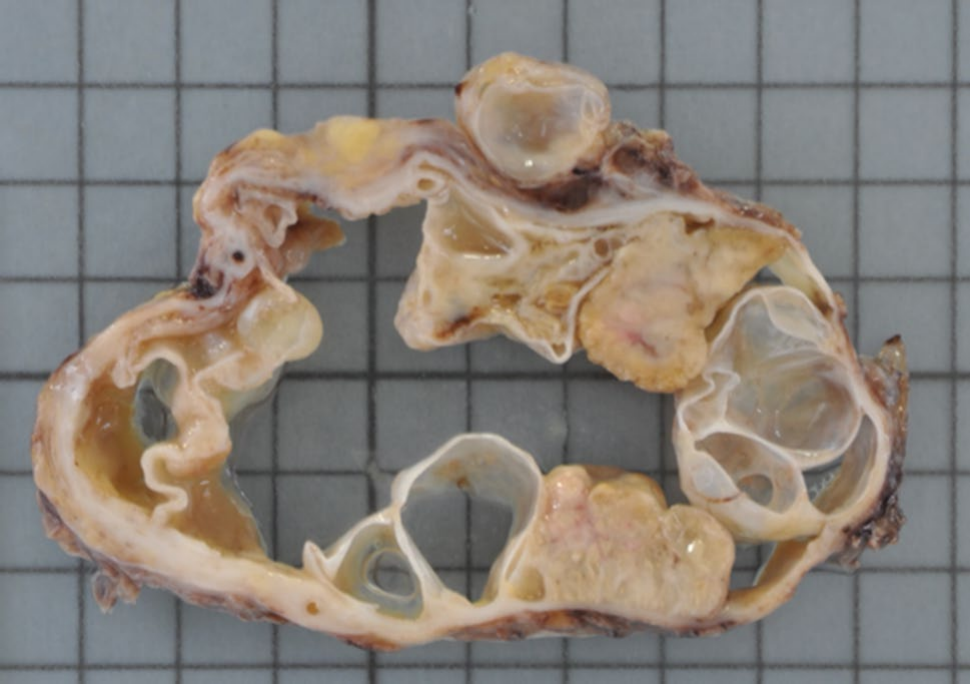
Specimen is a multilocular cystic tumor measuring 6.0 × 9.0 × 5.5 cm that is filled with pale yellow mucus. The multilocular cyst contains a yellowish ‐ white solid component with a maximum size of 2 cm

**Fig. 5:**
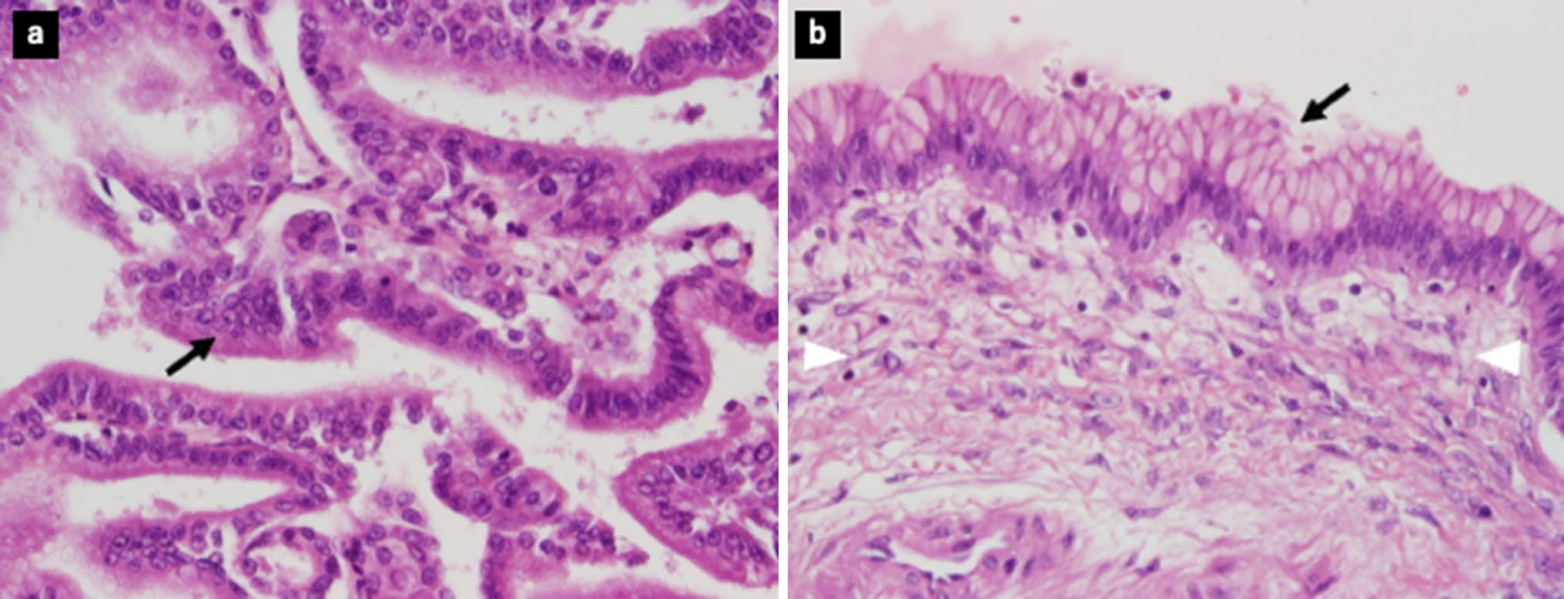
Histopathological findings with hematoxylin–eosin staining (400 ×) shows the existence of an ovarium stroma (white arrowhead) and high columnar epithelium with moderate dysplasia and mucus in the cytoplasm (black arrow). **a** Mural nodule. **b** Cyst wall

Distal pancreatectomy and lymph node dissection were performed for curative resection in July 2013. The cyst adhered to the spleen and stomach, and the spleen and part of the serosa were resected together. The peritoneal dissemination node was not revealed in the laparotomy findings, and cleaning of the peritoneal cavity was performed using saline. Peritoneal lavage cytology revealed adenocarcinoma. The specimen was a multilocular cystic tumor measuring 6.0 × 9.0 × 5.5 cm that was filled with pale yellow mucus (Fig. 4). The multilocular cyst contained a yellowish white solid component, with a maximum size of 2 cm. Although the main pancreatic duct was compressed and narrowed, there was no clear communication between the cyst and main pancreatic duct. Furthermore, rupture of the cyst capsule was unclear. Histopathological examination with hematoxylin–eosin staining showed the existence of ovarian stroma and high columnar epithelium with moderate dysplasia and mucus in the cytoplasm (Fig. 5). Except for ascites, no residual tumor was observed macroscopically or microscopically. No tumor cells were found in the dissected lymph nodes, spleen, or serosa of the stomach. Finally, the tumor was diagnosed as a non-invasive MCAC.

After surgery, she was treated with 120 mg/day of S-1 from days 1 to 28, repeated every 6 weeks per course, for four courses, as adjuvant therapy. She has been free of recurrence, including the no reappearance of ascites for more than 8 years after adjuvant therapy with S-1.

## Discussion

MCAC has been reported to have a better prognosis than typical pancreatic ductal adenocarcinoma [10]. However, even in MCAC with relatively favorable prognosis, there have been no previous reports of a case with malignant ascites, which resulted in surgical resection with a better prognosis, as in this report. Pancreatic MCN, including MCAC, is a rare pancreatic tumor, and the report of MCN with ascites is even rarer [11, 12]. MCN has a thick fibrous capsule, and rupture is very rare. However, five previous cases of MCAC with spontaneous rupture have been reported [13–17] (Table. 1). The longest prognosis of the five cases was 3 years. Consequently, the more than 8 years observed in this case is the longest prognosis, when the case was demonstrated to have ruptured. There are three reasons why this case could be considered as MCAC with rupture: (i) the cytology of the ascites was consistently adenocarcinoma, but no disseminated node was observed in both staging laparoscopy and laparotomy findings, and the final pathological diagnosis was non-invasive MCAC; (ii) epigastric pain suggestive of rupture was observed; and (iii) the size of the tumor was large. As for the symptoms, previous reports of MCAC rupture demonstrated symptoms suggestive of the onset of rupture, which may explain the epigastric pain in this case. The median sizes of MCN and MCAC have been reported to be 5 cm and 5.5 cm, respectively [4, 18], while the median size of past MCAC with rupture of 11.0 cm (range: 6.0–20.0 cm) [13–17], appeared larger than the median sizes of MCN and MCAC. The size in this case of 9.0 cm in diameter, does not also contradict the tendency of the past cases of MCAC with rupture. In this case, because the tumor cells had only moderate dysplasia and the malignant potential was considered to be low, it can be assumed that the tumor cells did not colonize the peritoneum, although the cystic tumor ruptured.

**Table 1:** Reported cases of ruptured MCAC

Authors	Age (years)	Sex	Tumor size (mm)	Location	Cytology of ascites	Peritoneal disseminated nodes	Surgical procedure	Follow-up (months)	Recurrence	Outcome
Smithers et al. [14]	33	Female	100	Body/tail	Unknown	No	DP	Unknown	Unknown	Unknown
Ozden et al. [13]	32	Female	150	Body/tail	Unknown	No	SPDP	12	No	Alive
Bergenfeldt et al. [15]	42	Female	200	Body	No malignancy	No	DP	19	No	Alive
Naganuma et al. [16]	32	Female	110	Head	Unknown	No	PD	36	Yes	Alive
Imoto et al. [17]	69	Female	60	Body/tail	Unknown	No	DP	2	No	Alive
Our case	28	Female	90	Tail	Adenocarcinoma	No	DP	102	No	Alive

*MCAC* mucinous cystadenocarcinoma, *DP* distal pancreatectomy, *SPDP* spleen-preserving pancreatectomy, *PD* pancreaticoduodenectomy

Standard chemotherapy for MCAC has not been established; thus, it has been treated as pancreatic ductal adenocarcinoma. Chemotherapy with GEM for unresectable MCAC has been successful in some reports, but not in others [6, 8]. This case was judged to be unresectable by staging laparoscopy, and GS, one of the treatment options for unresectable pancreatic ductal adenocarcinoma in Japan at that time [19], was initiated. In this case, chemotherapy with GS was retrospectively interpreted as preoperative adjuvant therapy. Currently, GS, a chemotherapy regimen, has been shown to be effective and safe as a preoperative adjuvant therapy for pancreatic ductal carcinoma [20]. Although the therapeutic effect of GS cannot be evaluated, since a PET–CT was not performed before its initiation, GS may be considered to have a certain antitumor effect given the decrease in CA19-9 levels. In the recent case report, the response by GEM plus nab-paclitaxel (GnP) and modified FOLFIRINOX (mFFX) (5-fluorouracil, leucovorin, irinotecan, and oxaliplatin) for unresectable MCAC was reported [9]. In addition to the GS used in this case, GnP and mFFX are also expected as chemotherapy regimen for MCAC in future.

If malignant ascites is present, surgical resection is not indicated for pancreatic ductal adenocarcinoma. Consequently, at the beginning, the cancer board of our institution judged this case as not an indication for surgical resection, due to the presence of malignant ascites. However, the risk–benefit balance of the treatment option continued to be examined during chemotherapy with GS, and finally, surgical resection was performed. There have been no previous reports of surgery performed in patients with MCAC, who have been preoperatively proven to have malignant ascites. However, the very good prognosis in this case suggests that surgical resection should be considered in the absence of peritoneal disseminated nodes on PET–CT and other imaging or staging laparoscopy findings, with careful follow-up after surgery. In fact, in this case, it seems to be also possible to carry out the upfront surgical resection without initiating chemotherapy, because the disseminated node has not been identified from the beginning. When the existence of peritoneal dissemination cannot be ruled out and there is a concern about an upfront surgical resection, it is important to administer chemotherapy, such as GS, as preoperative adjuvant therapy and to reassess the need for surgical resection based on imaging evaluation after a certain period.

## Conclusions

Surgical resection should be considered even in patients with MCAC with malignant ascites, as long as peritoneal disseminated node is not identified. If peritoneal dissemination cannot be ruled out and there is a concern about upfront surgical resection, continued evaluation of the feasibility of surgery during chemotherapy, such as GS, may be considered as a treatment strategy for MCAC with malignant ascites.
